# Immunotherapy: Shifting the Balance of Cell-Mediated Immunity and Suppression in Human Prostate Cancer

**DOI:** 10.3390/cancers4041333

**Published:** 2012-12-11

**Authors:** Jo A. Tucker, Caroline Jochems, James L. Gulley, Jeffrey Schlom, Kwong Y. Tsang

**Affiliations:** 1 Laboratory of Tumor Immunology and Biology, Center for Cancer Research, National Cancer Institute, National Institutes of Health, Bethesda, MD 20892, USA; E-Mails: joanne.tucker@nih.gov (J.A.T.); jochemscm@mail.nih.gov (C.J.); gulleyj@mail.nih.gov (J.L.G.); tsangkwo@mail.nih.gov (K.Y.T.); 2 Medical Oncology Branch, Center for Cancer Research, National Cancer Institute, National Institutes of Health, Bethesda, MD 20892, USA

**Keywords:** immunotherapy, prostate cancer, anti-tumor response, cell-mediated immunity

## Abstract

Active immunotherapy is dependent on the ability of the immune system to recognize and respond to tumors. Despite overwhelming evidence to support a cell-mediated immune response to prostate cancer, it is insufficient to eradicate the disease. This is likely due to a high level of suppression at the tumor site from a variety of sources, including immunosuppressive cells. Immune cells entering the tumor microenvironment may be inhibited directly by the tumor, stromal cells or other immune cells that have been induced to adopt a suppressive phenotype. The resurgence of interest in immunotherapy following the approval of sipuleucel-T and ipilimumab by the Food and Drug Administration has brought about new strategies for overcoming tumor-mediated suppression and bolstering anti-tumor responses. Improved understanding of the immune response to prostate cancer can lead to new combination therapies, such as the use of vaccine with small molecule and checkpoint inhibitors or other immunotherapies.

## 1. Introduction

Prostate cancer is the most prevalent cancer among U.S. men, and although the number of deaths from prostate cancer has been on the decline, it remains the second leading cause of cancer-related deaths in this population [1,2]. Localized prostate cancer can be treated with surgery or radiation, but about 30% of men develop recurrent disease. Recurrent or metastatic disease can often be controlled with androgen deprivation either through physical or chemical castration [3]. However, many patients become refractory to this treatment, developing castration-resistant prostate cancer (CRPC). Until recently, primary treatment for metastatic CRPC (mCRPC) consisted only of chemotherapeutic options. Now the U.S. Food and Drug Administration (FDA) has approved a therapeutic vaccine as a front-line treatment for mCRPC.

Prostate cancer presents an attractive model for immunotherapy based on several characteristics [4]. First, it is a relatively slow-growing tumor and second, it can be easily monitored by measuring serum levels of prostate-specific antigen (PSA). In addition to monitoring disease, the overexpression of PSA in prostate cancer serves as a target for immunotherapeutics. Prostate cancer also has several other well-described tumor-associated antigens (TAAs), including prostate-specific membrane antigen (PSMA) and prostatic acid phosphatase (PAP) [4]. As all of these antigens are tissue-specific, there is a decreased likelihood of any off-target side effects. In addition to these well-characterized antigens, several other novel TAAs overexpressed in prostate cancer have been described and evaluated as therapeutic targets. Prostate stem-cell antigen (PSCA) is overexpressed on the surface of primary androgen independent prostate tumor cells, as well as metastases [5]. Vaccination with dendritic cells loaded with a PSA/PSCA peptide has been shown to efficiently induce antigen-specific T cells *in vitro*, and in a Phase I/II clinical trial led to the complete regression of lymphadenopathy in 1/12 patients and stable disease in 6 others [6]. A peptide from the plasma membrane associated isoform of new gene expressed in prostate (NGEP) has been used to generate antigen-specific T cells *in vitro* that were capable of lysing NGEP-expressing human tumor cells [7]. Moreover, prostate cancer patients receiving a PSA-based vaccine had an increased frequency of NGEP-specific T cells post-vaccination. Another interesting prostate TAA is SLC45A3 (prostein). A common gene rearrangement in prostate cancer results in the formation of a fusion of prostein with the transcription factor ERG [8]. A prostein epitope was found to be capable of generating T cells that could kill prostate cancer cell lines [9], and a recent study reports that the loss of prostein correlated with gene rearrangement and shorter PSA-free survival time [10].

The presence of an immune response to prostate cancer can be seen in the form of tumor infiltrating lymphocytes (TILs) [11], particularly CD8^+^ T cells, which have been shown to be a positive prognostic factor in this disease and others [12,13,14]. However, cell-mediated anti-tumor responses are generally weak and inconsistent. This is likely because most TAAs are poorly immunogenic, in combination with a high level of immune suppression from the tumor and surrounding microenvironment. Utilizing the power and specificity of the immune system to fight tumors requires overcoming this inhibition to mount an effective response. The efficacy of active immunotherapies, such as therapeutic vaccines, may be improved by combining vaccines with treatments designed to alleviate suppression.

## 2. Cell-Mediated Immune Response to Prostate Cancer

As a component of the genitourinary tract, the prostate is part of the mucosal immune system. Prostate-associated lymphoid tissue is populated by T cells, natural killer cells (NK), dendritic cells (DC) and B cells, and is organized into two regions. The intraepithelial region consists of CD3^+^ T cells, predominantly CD8^+^, as well as NK, DC and B cells. The lymphoid aggregates form below the epithelial layer, arranged as B cell follicles, with parafollicular areas composed of mostly CD4^+^ T cells and DCs [15]. Prostate tumors contain infiltrates of both effector and suppressor cell types, including T, B, NK, macrophages and regulatory T cells [16]. This infiltrate was shown to be hormonally regulated as patients treated with androgen deprivation therapy (ADT) had significant increases in the density of CD3^+^ (*p* < 0.001) and CD8^+^ T cells (*p* < 0.001), and CD68^+^ macrophages (*p* < 0.001), as compared to patients receiving prostatectomy only. While a higher NK density correlated with lower risk of progression, a high density of macrophages was associated with risk of biochemical recurrence. Conversely, DC numbers have been reported to be significantly lower in prostate cancer than normal prostate tissue [17]. As DCs are primarily antigen presenting cells (APCs), a decrease in number could contribute to a lack of tumor-infiltrating lymphocyte activation. B cells can also act as APCs. Although intratumoral B cell numbers are not associated with clinical outcome [18], they could be acting as APCs in the absence of DCs [19].

### 2.1. T Cells

T cells, especially CD8^+^ cells, have long been thought of as the dominant mediators of anti-tumor activity for their recognition of endogenous peptides via HLA Class I expression. IFNγ release by T cells also plays an important part by upregulating Class I antigen processing and presentation in tumor cells [20]. This is supported by the increased incidence of tumors in immunocompromised patients, particularly those with T cell deficits, such as AIDS or transplant patients [21]. Compared to normal prostate, the density of infiltrating immune cells in benign prostatic hyperplasia (BPH) is significantly higher and composed of 70 to 80% T cells [22]. However, these numbers return to nearly normal levels in high-grade prostatic adenocarcinoma. A study by Ebelt *et al.* shows the formation of lymphocyte clusters near cancerous tissues, but few tumor-infiltrating cells [23]. The majority of CD3^+^ cells in both of these areas were CD4^+^ and CD69^+^. There was also a noted decrease in staining of both IFNγ and perforin in cancer tissue as compared to healthy prostate. TCR-Vβ analysis revealed a repertoire similar to that of normal prostate, indicating that there is an early T cell response to prostate cancer, but it appears nonspecific and dominated by CD4^+^ cells. Although these cells display the activation marker CD69, they do not appear to be functional, and therefore are unlikely to prevent tumor growth. Elsässer-Beile *et al.* reported that CD3^+^ TILs isolated from prostate carcinomas express significantly higher levels of IFNγ mRNA than those isolated from BPH [24]. This is in contrast to the previously mentioned decrease in IFNγ in carcinomas as measured by immunohistochemistry, possibly indicating a defect in protein production and thus impaired effector function.

In contrast to αβ T cells, γδ T cells do not kill in an antigen dependent manner, but have been shown to infiltrate some solid tumors and have lytic activity against cancer cells of epithelial origin [25]. Although little is known of the role of γδ T cells in human tumors, they have been shown to be protective in the TRAMP model of prostate cancer [26]. These cells can be expanded *in vivo* and have been reported to have antitumor activity against human prostate cancer cell lines *in vitro* [27].

### 2.2. NK and NKT Cells

Despite the obvious importance of T cells, NKs also contribute considerably to anti-tumor immunity. Gannon *et al.* report that prostate cancer patients receiving ADT had an elevated frequency of NKs, which correlated with a lower risk of progression (*n* = 75, *p* < 0.05) [16]. NKs were the predominant infiltrating cells in a patient exhibiting spontaneous regression of metastatic pancreatic cancer. Once isolated, these cells were shown to lyse autologous tumor and both pancreatic and prostate tumor cell lines [28]. The anti-tumor activity of NKs against prostate cancer has also been reported to increase with the introduction of reovirus and Lactobacillus [29,30].

NKT cells, which express both T cell and NK receptors, can also play a role in tumor immunity. Although most solid tumors do not express the NKT target molecule CD1, NKT cells can indirectly promote or suppress the antitumor response by influencing myeloid cells, such as DCs and macrophages [31]. A positive correlation was found between levels of NKT cells and CD8^+^ T cells in the peripheral blood of 54 prostate cancer patients, supporting the regulatory role of these cells in human tumor immunity [32].

## 3. Cell-Mediated Suppression of the Anti-Tumor Response

Although tumors use many mechanisms to escape immune recognition, cell-mediated inhibition is particularly nefarious as it effectively turns the immune system against itself. Responding cells infiltrating the tumor site may be induced to adopt a suppressive phenotype either directly through contact with the tumor or indirectly by secreted factors and other tolerogenic immune cells within the tumor microenvironment. Inhibitory cells include regulatory T cells (Tregs), myeloid-derived suppressor cells (MDSCs) and tumor-associated macrophages (TAMs).

### 3.1. Regulatory T cells

Regulatory T cells are potent mediators of inhibition and can be induced at, or recruited to, the tumor site by TGF-β and CCL22 signaling [33,34,35,36,37]. As seen with many tumor types, the peripheral blood and tumor of prostate cancer patients are enriched for Tregs [38,39,40]. Yokokawa *et al.* previously reported that Tregs isolated from the peripheral blood of prostate cancer patients had increased suppressive function compared to healthy individuals (prostate cancer patients, 55.7 + 18.92%; healthy donors, 31.2 + 6.55%) [41]. Data also suggest that a decrease in Treg function post-vaccination with PSA-TRICOM may be associated with increased overall survival in mCRPC [42,43].

The induction of Tregs and T cell tolerance in tumors is likely tumor- or DC-mediated via molecules such as PD-1 and TGF-β [33,44,45]. Inflammatory factors at the tumor site promote tolerance and inhibit maturation of DCs, which in turn, may induce an immunosuppressive or tolerogenic phenotype in both naïve and effector T cells [11,46,47]. TGF-β signaling induces CD4^+^ T cells to produce prostaglandin E_2_ (PGE_2_), which contributes to Treg generation [48] and inhibits the ability of DCs to attract naïve T cells via CCL19 production [49]. It has been previously reported that patients with localized prostate cancer showed increased serum levels of PGE_2_ compared to patients with metastatic or biochemically progressive disease, or healthy donors [41]; this increase positively correlated with Treg function (R = 0.814). Treatment with 13, 26, 52 or 100 μM dimethyl PGE_2_ enhanced the suppressive function of Tregs from healthy donors and patients with biochemically progressive or localized, but not metastatic, prostate cancer, ranging from a 1.6-fold increase at 13 μM to a 2.4-fold increase at 100 μM. This phenomenon is supported by another study in prostate cancer reporting that prostatectomy restored DC maturation and decreased Treg frequency in tumor-bearing patients [50]. Androgen deprivation therapy has been shown to increase Treg infiltration of the prostate; however, as CD8^+^ cytotoxic lymphocytes increased by the same ratio, no impact on disease-free survival was seen [51].

Several treatments aimed at depleting Tregs have been developed and tested in mouse models of prostate cancer [52]. However, as Tregs are not characterized by a specific cell surface marker, these methods are non-specific and often target activated lymphocytes as well. Human studies have focused on immunotoxins fused with either anti-CD25 antibodies or IL-2 [53,54,55]. Although some benefits have been reported, the effect seems to be transient as Tregs continue to be induced and quickly return to pre-treatment levels. Combining Treg depletion with vaccination seems promising, but will require optimizing dosage and scheduling in order to be effective. Another method of targeting Tregs is blockade of function. Ipilimumab, a monoclonal antibody against the activation marker CTLA4, has been extensively tested in humans and will be discussed at length below.

### 3.2. Myeloid-Derived Suppressor Cells

Myeloid-derived suppressor cells comprise various stages of differentiation along both the monocytic and granulocytic lineages, which may indicate that they can be induced at various early stages of development or that they originate from an early precursor [56,57]. Like Tregs, MDSCs accumulate in the peripheral blood of cancer patients [58]. Removal of these cells restored the ability of the DC fraction to activate allogeneic T cells *in vitro*. When isolated, MDSCs could not be matured to DCs, but were able to suppress T cell proliferation [58]. Cancer patients receiving standard treatment had a much higher percentage of MDSCs than either age-matched controls or untreated patients; this increase correlated with clinical cancer stage and metastatic tumor burden [58,59]. This indicates that the accumulation of MDSCs in cancer patients may be driven by the tumor, but can also be influenced by chemotherapies, such as cyclophosphamide. Thus, MDSCs represent another powerful mediator of immune suppression in prostate cancer patients, but due to their heterogeneous nature, may prove difficult to target for depletion.

### 3.3. Tumor-Associated Macrophages

Although TAMs are also of the myeloid lineage, they are a fully differentiated cell type. These resident macrophages act as part of the tumor stroma by promoting tumor growth and progression. Like other suppressive cells, TAMs are polarized to an M2 or tolerogenic phenotype by tumor-secreted factors [60,61]; however, there is evidence that their functions may differ based on location within or around the tumor (reviewed by Mantovani *et al.* [62]). In contrast to the classical M1 macrophages, which are efficient effectors, antigen presenters and produce T cell stimulating cytokines, M2 macrophages are beneficial to tumor growth and survival as they have poor antigen presenting ability, produce both pro-angiogenic and immunosuppressive factors, and promote tissue remodeling [63,64]. In human prostate cancer, increasing TAM size, number and area of infiltration were found to correlate with increasing Gleason score, but there is some disparity between studies as to the value of TAM quantification as a prognostic marker in this disease [65,66]. However, Satoh *et al.* showed that co-injection of mice with human prostate tumor cells and macrophages overexpressing IL-12 enhanced expression of MHC in TAMs increased T cell infiltration and reduced both the primary tumor and lung metastases [67]. TAMs not only suppress the immune response, but also directly support tumor growth, thus becoming an important factor for consideration in the development of future immunotherapies.

## 4. Immunotherapy for Prostate Cancer

Several different modes of immunotherapy for prostate cancer are currently being investigated in clinical trials. The mechanism of action for these therapies varies, aiming either to activate the immune system to better recognize and kill tumor cells, or to decrease suppression of the immune cells. Clinical trials combining therapeutic cancer vaccines with chemotherapy, radiation, and small molecule targeted therapeutics are in progress and may provide a new alternative for the treatment of prostate cancer. 

One target of small molecule inhibitors that seems to be particularly promising for prostate cancer is c-Met. The c-Met pathway is dysregulated in many human cancers, including prostate, and is often associated with poor clinical outcome and shortened survival [68,69]. In prostate, c-Met signaling is mediated in a paracrine manner. Its ligand, hepatocyte growth factor (HGF), can be produced by stromal cells around the prostate, but also in bone, making c-Met a good target for localized and metastatic disease [70]. Expressed on a variety of tissues and organs, c-Met is mainly found on epithelial cells, but can also be expressed on monocytes and DCs [71]. Ligand activation of c-Met regulates the migration, but not the antigen presenting function, of DCs. Although further characterization of the immune response to c-Met blockade is needed, treatment with small molecule inhibitors to such targets should be a good candidate for combination with immunotherapy. Several c-Met inhibitors are in phase I or II clinical trials for prostate cancer and promising early results have been obtained with at least one, XL184 (cabozantinib) [72].

ADT, which is the first line of treatment for prostate cancer, has also been shown to affect the immune environment in several ways (reviewed by Aragon-Ching *et al.* [73]). T cell and macrophage infiltration of the prostate [16,74], and thymic regeneration with increasing T cell numbers, have been shown in humans after ADT [75]. Increasing the number of T cells may have a positive impact on immunotherapy. The following discussion highlights some of the recent advances and most promising immunotherapies for prostate cancer.

### 4.1. Sipuleucel-T (Provenge®)

Sipuleucel-T consists of autologous antigen-presenting cells that have been enriched and pulsed with a fusion protein of GM-CSF and PAP [4]. This product is given at three time points over the period of a month. Based on a phase III study in 512 patients, which showed increased overall survival (25.8 *versus* 21.7 months, *p* = 0.01) [76], the FDA approved sipuleucel-T for asymptomatic or minimally symptomatic mCRPC. The immune response to sipuleucel-T in patients was generally evaluated 2–3 months after initiation of therapy, and although median time to progression was 10–11 weeks [77], phase I/II trials reported significant T cell proliferation response to the target antigen, some decline in PSA, as well as evidence of antigen-specific T cell generation and antibody production [78,79]. Of 31 patients tested, 100% developed T cell proliferation responses upon three infusions with sipuleucel-T, after having little or no reaction pre-vaccination [78]. Serum PSA decreases were reported in six patients, three of whom experienced serum PSA decreases greater than 50%. The median time to disease progression was significantly greater for patients who developed an immune response (34 weeks, n = 20) than for those who did not (13 weeks, n = 11) (*p* < 0.027). A significant correlation between antigen-specific immune responses and overall survival (OS) (*p* = 0.003) was reported in a combined analysis of three phase III trials of men with mCRPC [80]. Although only antibody response independently correlated to OS, antigen-specific cellular responses were seen in 60% of patients receiving sipuleucel-T by T cell proliferation assay and 48% in IFNγ ELISPOT, while responses in controls subjects were 6% and 13%, respectively.

### 4.2. PROSTVAC®-VF

PROSTVAC is a poxviral vaccine (using a vaccinia vector for priming and fowlpox vector for boosting) containing PSA and three costimulatory molecules (B7.1, ICAM-1 and LFA-3, designated TRICOM) [81]. It is administered subcutaneously with GM-CSF, given on the same day as the vaccine and for three consecutive days. Recently, a multicenter randomized controlled phase II study of PROSTVAC in men with asymptomatic or minimally symptomatic mCRPC showed a 44% reduction in death rate and an 8.5 month improvement in overall survival compared to patients treated with control vectors [76]. Post-vaccination, 12/32 patients had decreases in serum PSA and 2/12 patients with soft tissue disease had decreases in index lesions [43]. Patients who had greater PSA-specific T cell responses by ELISPOT also showed a trend toward longer survival (*p* = 0.055), and 12/15 patients evaluated lived longer than predicted by the Halabi nomogram (*p* = 0.035) [82].

Human Tregs are very heterogeneous phenotypically, but it has been demonstrated that the most active of them express high levels of CTLA4 [83]. Although there was no difference in CD4^+^CD25^+^CD127^−^FoxP3^+^ Treg levels pre- *versus* post-vaccination with PROSTVAC, there was a significant increase in the effector to CTLA4^+^ Treg ratio in patients with longer than predicted survival and a significant decrease in patients with shorter than predicted survival [42]. Likewise, Treg suppressive function decreased post-vaccination in 10/13 patients with longer than predicted survival and increased in 6/8 patients with shorter than predicted survival [41]. Vergati *et al.* also found a significant correlation between the percentage of CTLA4^+^ Tregs and suppressive function, and that patients with shorter than predicted survival had a significantly higher percentage of CTLA4^+^ Tregs post-vaccination (*p* = 0.019) [42]. This study supports the evidence that patients with less disease burden may receive the most benefit from vaccine immunotherapy and that vaccination can have an impact on overall survival in prostate cancer patients. An international randomized controlled phase III trial of PROSTVAC opened in 2011 (NCT01322490) [84]. 

### 4.3. Ipilimumab (Yervoy™)

Ipilimumab is a fully humanized antibody that binds to cytotoxic T lymphocyte-associated antigen 4 (CTLA4) on activated T cells, preventing inactivation of these cells [85]. CTLA4 is also constitutively expressed on Tregs; thus blockade could alleviate Treg-mediated immune suppression [86]. However, Kavanagh *et al.* reported that treatment with anti-CTLA4 antibody resulted in an increase in both effector CD4^+^ T cells and functional Tregs in the periphery [86]. Ipilimumab has been approved for first and second line treatment of melanoma, and is currently being investigated in phase III trials for prostate cancer and phase II trials for non-small cell lung cancer. In a phase I trial for mCRPC, the combination of ipilimumab with GM-CSF resulted in a higher frequency of activated circulating CD8^+^ T cells and antibody responses to TAAs [87]. In the cohort receiving the highest dose, 50% (3/6) of patients had a PSA response and there was one partial response in visceral metastases. In a phase II trial in which ipilimumab was given with or without a single dose of docetaxel, 6/46 patients showed a PSA decline [88]. Along with anti-tumor responses, there have also been several immune-mediated adverse events reported with ipilimumab treatment [89,90]. However, in a small pilot trial of 14 mCRPC patients, a relatively low dose of ipilimumab (3 mg/kg) was well-tolerated and resulted in PSA declines of ≥50% in two patients [91]. Ipilimumab is currently being used in combination with vaccines and other drugs in several clinical trials. A clinical trial examining the combination of ipilimumab with GVAX®, a GM-CSF transfected allogeneic tumor cell vaccine, reported a median overall survival of 29.2 months (95% CI 9.6–48.8 months), while ipilimumab in combination with PROSTVAC® yielded a median overall survival of 34.4 months (95% CI 29.6–41 months) [92,93]. Both studies reported toxicities similar to treatment with ipilimumab alone. Although these were phase I dose-escalation trials, a greater than 50% decrease in PSA after treatment was reported in seven of 28 chemotherapy-naïve patients in the GVAX® trial and in six of 24 patients in the PROSTVAC® trial, thus warranting further research on the combination of ipilimumab and vaccine.

## 5. Conclusions

Although the human immune system is capable of recognizing and mounting a response to prostate cancer, this response is often circumvented by tumor-derived inhibition. This inhibition may include the induction of cells to a suppressive phenotype, effectively turning the immune response against itself. Despite recent developments and some limited successes in immunotherapy, it has yet to fulfill its potential. A multifaceted approach that combines vaccine with targeted therapies, such as small molecule and checkpoint inhibitors, may prove more successful by not only directing an effective immune response but also breaking the cycle of inhibition promoted by the tumor.
